# Multiple primary synchronous malignant tumors

**DOI:** 10.1186/s13104-015-1724-5

**Published:** 2015-11-27

**Authors:** Alberto Testori, Ugo Cioffi, Matilde De Simone, Francesco Bini, Adriano Vaghi, Alessandro A. Lemos, Michele M. Ciulla, Marco Alloisio

**Affiliations:** Department of General and Thoracic Surgery, Humanitas Research Hospital, via Manzoni 56 Rozzano, 20089 Milan, Italy; Depatment of Surgery, University of Milan, Milan, Italy; Department of Pneumology, “G. Salvini” Hospital, Garbagnate Milanese, Milan, Italy; Department of Diagnostic Radiology, Princess Elisabeth Hospital, Channel Islands, GY4-6UU UK; Laboratory of Clinical Informatics and Cardiovascular Imaging, Department of Clinical Sciences and Community Health, University of Milan, Milan, Italy; Cardiovascular Diseases Fondazione IRCCS Cà Granda Ospedale Maggiore Policlinico, Milan, Italy

**Keywords:** Multiple primary malignancies, Synchronous tumors, Metachronous tumors

## Abstract

**Background:**

Patients with primary multiple malignancies are progressively increasing due to prolonged survival of cancer patients and to the advances in diagnostic techniques and therapeutic options.

**Case presentation:**

Here we present a 66 year-old caucasian patient with four synchronous primary malignant tumors affecting the lung, oropharynx, large bowel and prostate gland, respectively, treated with multidisciplinary approach.

**Conclusions:**

The increased incidence of multiple malignant tumors is a real challenge to the clinician and clinical attention should be made to avoid a misdiagnosis. In addition an early diagnosis is essential to achieve a radical treatment. We believe that the treatment modality should be carefully made and tailored on the individual patient suffering from this disease.

## Background

Patients with multiple primary malignancies (MPMs) are progressively increasing; these tumors may be metachronous or synchronous. This distinction implies important diagnostic and therapeutic challenges. From a diagnostic point of view the different patterns of MPMs should be considered. Therapeutically, a multi-disciplinary and patient-oriented approach should be considered. Hereby, we present a case of four primary malignant synchronous tumors affecting the lung, oropharynx, large bowel and prostate gland, respectively.

## Case presentation

**Fig. 1:**
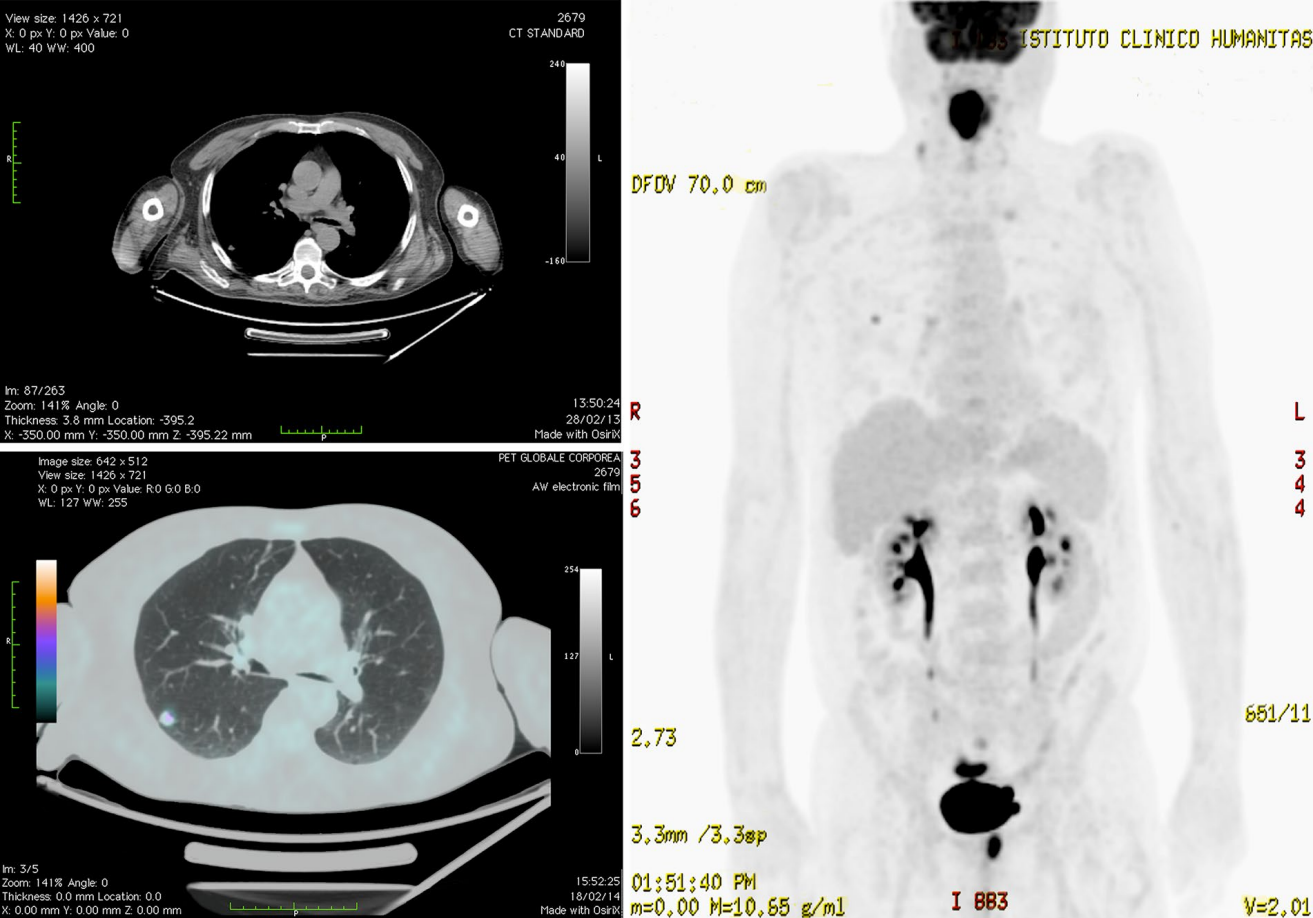
*Top left panel* Axial non-contrast CT scan shows no significant mediastinal, hilar and axillary lymphadenopathy. *Bottom left panel* Axial non-contrast lung window setting CT shows a solid nodule at the upper segment of right lower lobe. *Right panel* Coronal view whole-body PET scan shows focal uptake of at the upper segment of right lower lobe, sigmoid colon and prostate gland

A 66-year-old male was referred to our department because of cough, chest pain and weight loss. His past clinical history, family history were unremarkable. Given the persistency of symptoms, chest X-ray was performed and showed a subtle opacity at the upper segment of the right lower lung. Whole body computed tomography (CT) scan confirmed the presence of a pulmonary malignant-looking nodule without hilar lymphadenopathy. ^18^F-fluorodeoxyglucose positron emission tomography (FDG-PET) revealed avid uptake of the pulmonary nodule as well as oropharyngeal, sigmoid colon, and prostate gland uptake (Fig. 1). Subsequently, the patient underwent video-assisted bronchoscopy, which revealed normal findings. Conversely, video-assisted laryngoscopy showed an infiltrative ulcerated lesion involving the base and both valves of the tongue. Oropharyngeal biopsy was performed and histology revealed an infiltrative squamous cell carcinoma. Subsequently, CT guided lung biopsy showed a lung adenocarcinoma. The patient underwent colonoscopy with polypectomy and histology revealed the presence of adenocarcinoma. Finally, specimen from the prostate gland revealed an adenocarcinoma (Gleason score: 3 + 3), too (Table 1). Abnormally enlarged lymph nodes in the abdomen up to 1.7 cm in diameter along with several non-specific lymph nodes have been identified. CT scan of the neck and facial bones showed a bulky mass in the right aspect of the oral cavity, infiltrating the base of the tongue with preservation of the adjacent mandibular cortical bone abutting the midline, given a horseshoe-like appearance of the tumor. There was infiltration of the muscles of the tongue base whereas the left mylohyoid muscle was preserved. Bilateral enlarged lymph nodes at level II and III have been identified. Given the presence of these multiple malignant tumors, multidisciplinary assessment was necessary. The laryngeal lesion was treated by radio and chemotherapy whereas the sigmoid and prostate tumors were treated by surgical excision. In regard to the pulmonary tumor, the decision about whether surgery or radiotherapy would be more appropriate was considered later. Consequently, chemotherapy and radiotherapy were started given almost complete resolution of the lung tumor; instead we observed progression of the sigmoid tumor along with two enlarged lymph nodes in the pelvis, whereas the tumor of the prostate gland did not change in size. The patient had left hemicolectomy and prostatectomy, which confirmed the presence of adenocarcinoma with features of vascular invasion, adipose tissue invasion, and no extramural or perineural involvement. Metastases were found in 10 out of 19 lymph nodes. As a result, the histological staging was pT3 N2B R0 B. Prostatic specimen confirmed the presence of adenocarcinoma with no infiltration of the urinary bladder. However, multifocal extension by the tumor to the adjacent tissues was observed. A 30-day interval follow-up PET-CT scan showed an avid focal uptake at segment 5 of the liver suspicious of a sigmoid adenocarcinoma metastasis and at the apical segment of the right lower lobe in accordance with the known pulmonary tumor. A further multidisciplinary assessment regarding the appropriate patient’s management suggests surgical treatment for the pulmonary lesion, stereotactic radiotherapy for the metastatic deposit in the liver and adjuvant chemotherapy for the sigmoid tumor of colon. Surgical specimen after right lower lobectomy showed a G3 type lung carcinoma with prevalent aspects of acinar growth and absence of pleural infiltration, vascular invasion, or necrosis. The tumor did not involve the bronchial or vascular surgical resection margins or adjacent lymph nodes. Immunocytochemistry was TTF1 (+) and CDX2 (−), whereas histopathological staging was pT1 N0. The patient received stereotactic radiotherapy for the liver metastasis and adjuvant chemotherapy for the sigmoid colonic tumor. An 18-month interval follow-up PET-CT scan demonstrated no recurrence.

**Table 1:** Clinical history of the patient: diagnosis and treatment

Date (mm/dd/yrs)	Examination	Histology	Date (mm/dd/yrs)	Treatment
02/08/2013	Laringoscopy biopsy	Vegetating lesion base of the tongue *Infiltrating and ulcerating squamous cell carcinoma C2*–*C3*	From 03/12/2013 to 04/13/2013	2 cycles of TPF (taxotere/cisplatin/5-fluoruracil) plus rasdiation therapy on nodes PET + and loco regional (69.96 Gy) and on orofarinx (54.45 Gy)
02/08/2013	CT-guided pulmonary biopsy	Subpleural lesion o f the right upper lobe *Pulmonary Adenocarcinoma TTF1(*+*) e CDX2(*-*). pT1a N0*	09/30/2013	Right lower lobectomy No chemotherapy
02/13/2013	Pancolonscopy Polipectomy	Adenocarcinoma CDX2(+); TTF1(−). *pT3a N2b R0*	07/11/2013	Left hemicolectomy Adjuvant chemotherapy
03/07/2013	Prostatic biopsy	Adenocarcinoma *Grading Gleason score 4* + *3; pT3, N2b, R0B*	07/11/2013	Prostatectomy No chemotherapy

## Conclusions

Since the first report of Billroth and the definition of Warren and Gates [1], the incidence of multiple cancers had progressively increased over time. The first point that deserves clarification regarding multiple tumors is what does the term ‘‘primary’’ means. First, tumors must be histologically different. Second, they must involve different organs. Finally, metastatic lesions among these tumors must be excluded. MPMs are generally divided into 2 categories: metachronous, when tumors follow one another regardless a fixed period of time and synchronous, when tumors arise simultaneously or within 6 months from the primary malignant tumor [2]. Metachronous are more frequent than synchronous tumors with a ratio of 2.7: 1. Second primary tumors are most common, whereas third and fourth primary tumors are relatively rare [3]. There are several explanations for the origin of these tumors. One is the growing incidence of multiple tumors due to increased lifetime [4]. Another is that effective anti-neoplastic therapy has led to a significant improvement in patients’ survival from cancer. Therefore, survivors have a 20 % higher risk of new primary cancer in the same or different organs than the general population [5]. The tendency of some subjects to develop multiple tumors (synchronous or metachronous) may be explained either by an individual predisposition or by the action of carcinogenic factors acting on different organs at different times. This is probable the explanation regarding the association between low growing and aggressive tumors, as reported in our case. The pathogenesis of multiple and single tumors has similar mechanisms. The combined action of environment and genetic factors facilitates the onset of a new tumor. Therefore, multifactorial and predisposing factors are likely responsible for the development of metachronous tumors [3]. Conversely, it is difficult to explain the origin of synchronous tumors, even if multifactorial and predisposing factor cannot be exclude, their onset seems to be more time depending. Even if there are several limitations in the current literature because most are case-report studies, the reported incidence of metachronous and synchronous tumors is relatively high [3]. Therefore, radiologists and clinicians should be aware about different patterns and clinical presentation of multiple malignant tumors. In conclusion, successful patient’s management and increased life expectancy can be achieved by multidisciplinary management and patient-oriented approach in multiple primary malignant synchronous tumors.

## Consent

Written informed consent was obtained from the patient for publication of this case report and accompanying images.

## Abbreviations

MPMs: multiple primary malignancies
CT: computed tomography
FDG-PET: ^18^F-fluorodeoxyglucose positron emission tomography
PET-CT: positron emission tomography-computed tomography
TTF1: thyroid transcription factor-1
CDX2: caudal type homeobox transcription factor-2
